# Pityriasis Lichenoides et Varioliformis Acuta Case with Enterobiasis, Giardiasis, and *Helicobacter pylori* Infection

**DOI:** 10.5152/eurasianjmed.2023.23026

**Published:** 2024-02-01

**Authors:** Mehmet Melikoğlu, Büşra Solak Esen, Rabia Yamak, Ali İşlek

**Affiliations:** 1 Department of Dermatological and Venerial Diseases, Atatürk University Faculty of Medicine, Erzurum, Turkey; 2 Department of Pediatric Gastroenterology, Çukurova University Faculty of Medicine, Erzurum, Turkey

Pityriasis lichenoides (PL) is a rare disease group, consisting of pityriasis lichenoides chronica and pityriasis lichenoides et varioliformis acuta (PLEVA).^1^ Here, a case of PLEVA accompanied by *Giardia intestinalis, Enterobius vermicularis, and Helicobacter pylori* infections is presented, because there is no case associated with intestinal pathogens in the literature.

An 8-year-old male patient without known additional disease was admitted to our department with a complaint of rash that started 2 months ago. It was learned that the patient had previously applied to a different center with this complaint and received 4-week treatment; the lesions regressed almost completely and started to increase 1 week after the end of treatment. The patient had no history of drug use or infection. Dermatological examination revealed erythematous papules accompanied by crusts and scales, atrophic scars, and hypopigmented macules (Figure 1). The diagnosis of PLEVA was confirmed histopathologically. Because of the minimal decrease in ferritin and hemoglobin values in laboratory tests and the occasional description of abdominal pain, the patient consulted to the pediatrics. It was recommended to study occult blood in stool and to send stool parasite tests. Hemoglobin and transferrin were found positive twice in stool occult blood test. *G. intestinalis* cysts were detected in the microscopic examination of stool. Endoscopy and colonoscopy were planned. Endoscopy revealed findings consistent with pangastritis and *H. pylori* positivity. In colonoscopy, luminal pinworms (*E. vermicularis*) were observed in the cecum. Albendazole for enterobiasis and metronidazole for giardiasis and *H. pylori* eradication were started. Long-term remission was observed after treatments. Lesions except atrophic scars and postinflammatory hypopigmentations completely regressed at the patient’s second month follow-up. Written informed consent was obtained from the parents of the patient who agreed to take part in the study.

Pityriasis lichenoides is a rare disease group whose etiopathogenesis has not been clearly elucidated. Current studies define PL as a lymphoproliferative disease triggered by a possible antigenic stimulus such as viruses.^2^ Findings suggestive of infectious agents in the etiology of PL are autumn and winter onset, reports of familial outbreaks, disease remission with the treatment of the suspected pathogen, and detection of antigenic structures of viral antigens in skin biopsies by polymerase chain reaction.^2-4^ Topical steroids and systemic antibiotics are used successfully in the treatment of PLEVA.^5^ Pyrimethamine and antiviral agents were also tried and successful treatment results were obtained in PLEVA cases whose etiology was accused of viral agents.^3^ Studies conducted so far have mostly focused on viral agents and vaccines as precipitating factors in PLEVA. Pityriasis lichenoides et varioliformis acuta has ultimately been interpreted as a reaction initiated by the antigenic stimuli. In the examinations we performed *G. intestinalis* and *E. vermicularis* pathogens in the intestine and *H. pylori* positivity in endoscopy were found. In this case, which relapsed in a short time with the previous treatment, long-term remission was achieved with parasitic agent treatment.

Intestinal parasites and *H. pylori* may play a triggering role in PLEVA. Shortening of the recovery period of PLEVA cases can be achieved with treatments for the etiology. Further studies can be planned to determine the role of parasitic agents in PLEVA.

## Figures and Tables

**Figure 1.**
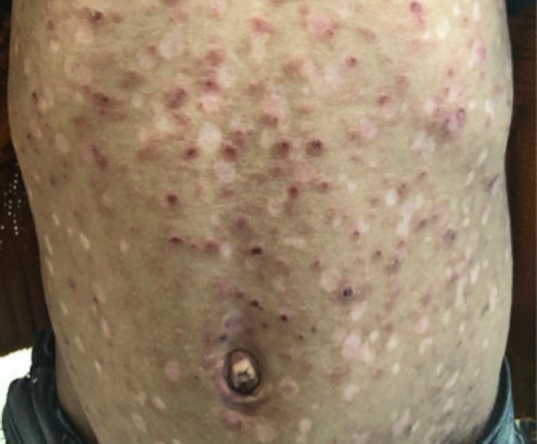
Lesions before treatment.
